# Efficacy and safety of metformin versus empagliflozin on chronic kidney disease progression (MET-EMPA-CKD): a randomized controlled trial

**DOI:** 10.1186/s13098-025-02040-9

**Published:** 2025-12-10

**Authors:** Bassant M. Mahboub, Ayman F. Refaie, Sahar M. El-Haggar, Yasser M. Hafez, Tarek M. Mostafa

**Affiliations:** 1 Drug and Poison Information Center, Faculty of Pharmacy, Tanta University, Tanta, 31527 Egypt; 2 Clinical Pharmacy and Pharmacy Practice Department, Faculty of Pharmacy, Mansoura National University, Gamasa, 7731168 Egypt; 3 Nephrology and Transplant Unit, Urology and Nephrology Center, Mansoura University, Mansoura, 35516 Egypt; 4 Clinical Pharmacy Department, Faculty of Pharmacy, Tanta University, Tanta, 31527 Egypt; 5 Internal Medicine Department, Faculty of Medicine, Tanta University, Tanta, 31527 Egypt

**Keywords:** Metformin, Empagliflozin, CKD progression, Renoprotection, TGF-β, KIM-1, Autophagy

## Abstract

**Background:**

Chronic kidney disease (CKD) is a devastating progressive condition accompanied with high morbidity and mortality rates. Sodium-glucose cotransporter-2 (SGLT2) inhibitors have recently proven their renoprotective effects, whereas evidence for metformin remains limited but suggestive of potential benefit. This study aimed at comparing the efficacy and safety of metformin versus empagliflozin, a SGLT2 inhibitor, on retarding CKD progression with exploring supposed mechanistic pathways in clinical settings.

**Methods:**

In this 12-month randomized controlled trial, 120 moderate CKD patients were randomized into three groups: metformin 1000 mg/day (*n* = 40) or empagliflozin 10 mg/day (*n* = 40), both added orally to standard treatment, or control who continued standard of care (*n* = 40). The primary outcome was changes in estimated glomerular filtration rate (eGFR). Secondary analyses assessed percent changes of urinary albumin-to-creatinine ratio (uACR), transforming growth factor-β1 (TGF-β1), kidney injury molecule (KIM)-1, and beclin-1 (an autophagy biomarker). Other metabolic and safety issues were also assessed.

**Results:**

118 patients completed the study with comparable baseline data. Metformin and empagliflozin halted the decline in eGFR at study end with adjusted mean difference ± SE: 8.91 ± 1.92 (*p*˂0.001) and 5.1 ± 1.89 (*p* = 0.03), respectively, compared to control group. Metformin preserved its effect in diabetics and non-diabetics, with superiority than empagliflozin in non-diabetics. uACR was lowered by metformin and empagliflozin than control. Both of them tended to halt the deterioration of intermediates with %relative change of -28.8% (95% CI, -44.4 to -9, *p* = 0.003) and 179.3% (95% CI, 32.2 to 490, *p* = 0.003), for metformin versus control in TGF-β1 and beclin-1 levels, respectively. Empagliflozin reduced KIM-1 compared to control [-29% (95% CI, -49.3 to -0.5, *p* = 0.045)]. Study treatments showed benefits on lipid profile without changing urate levels significantly compared to the control arm. No significant changes were found between metformin and empagliflozin. Adverse effects were comparable across groups with tolerable increased urination frequency by empagliflozin.

**Conclusion:**

12-month metformin therapy demonstrated renoprotective effects comparable to empagliflozin, with a greater effect observed among non-diabetics as an exploratory insight. Metformin’s renal actions were linked to antifibrotic and favorable autophagy effects while, empagliflozin preserved mainly tubular injury. Safety issues were generally comparable.

**Clinicaltrials.Gov identifier:**

NCT05373680, registered on 13/5/2022 “retrospectively”.

**Graphical abstract:**

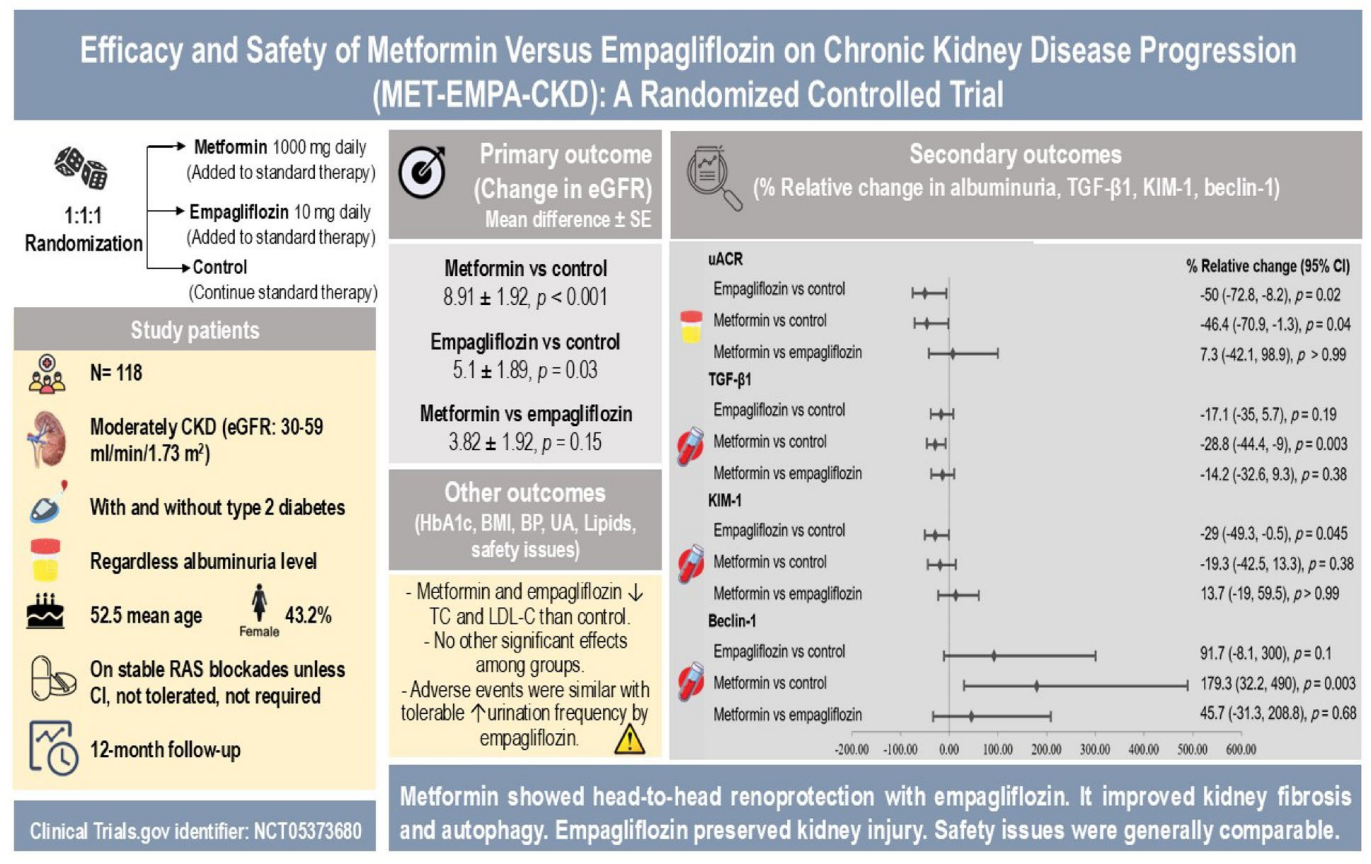

**Supplementary Information:**

The online version contains supplementary material available at 10.1186/s13098-025-02040-9.

## Introduction

Chronic kidney disease (CKD) is a major global health concern affecting nearly 850 million cases with a prevalence of 10% worldwide. It is estimated to rank the top fifth cause of death by 2040 [1]. In Egypt, CKD affected about 13% of its population in 2017 [2]. CKD exhibits a progressive damage of kidney tissues leading to a permanent, irreversible loss of their functions with the need for dialysis or renal transplant in such cases [3]. Hemodynamic, tubular injury, and endothelial dysfunction, modulated by nutritional status and lifestyle behaviors play critical roles in disease progression and patient outcomes [4–7]. Indeed, the pathophysiology of CKD involves cascaded pathways of inflammation, fibrosis, and apoptosis, with subsequent cellular dysfunction and disease aggression [3]. Transforming growth factor-β1 (TGF-β1) plays a crucial role in kidney fibrosis and acts as a potent stimulator for glomerulus mesangial cell hyperplasia. Upregulation of TGF-β1 is shown to be associated with renal function deterioration in CKD [3, 8]. On the other hand, autophagy, a conserved lysosomal pathway for the degradation of cytoplasmic components, addresses the maintenance of kidney homeostasis, structure, and function. Its dysregulation contributes to the pathogenesis of kidney injuries [9]. Levels of beclin-1, a key regulator of autophagy, are found to be decreased in CKD patients [10]. Additionally, kidney injury molecule (KIM)−1 is a specific marker for hypoxic injury to proximal tubular cells. It is incorporated in the pathogenesis of tubulointerstitial inflammation during chronic renal injury through the secretion of chemokines/cytokines with subsequent aggravating disease state [3].

Treatment of CKD addresses the management of predisposing causes, mainly diabetes and hypertension, with other supportive care or specific immunotherapy in some instances [11]. Despite attempts to achieve optimal glycemic and blood pressure control and the widespread use of renin-angiotensin system blockades, there remains a high residual risk for CKD progression mandating the presence of intended renoprotective therapies [12, 13]. Sodium-glucose cotransporter-2 (SGLT2) inhibitors are a relatively new anti-hyperglycemic therapeutic class. They have recently proven cardio- and reno-protective effects even in non-diabetics [14–16]. SGLT2 inhibitors activate tubulo-glomerular feedback by returning sodium in macula densa, hence they preserve afferent vasoconstriction and reduce intraglomerular pressure and hyperfiltration [17, 18]. Although, their clinical benefits are well proven, the intermediate mechanistic pathway is not fully understood.

Metformin, the most commonly used drug when addressing type 2 diabetes, possesses favorable pleiotropic beneficial effects. It was previously restricted to be used in diabetic nephropathy due to lactic acidosis (LA) risk in addition to its main renal elimination pathway [19]. However, further reports demonstrated the rare incidence of LA which could be triggered mainly by other predisposing conditions. In contrast, emerging evidence suggests renoprotective effects of metformin according to preclinical, isolated human tissue, and other observational clinical studies. At the same time, controversial findings have raised debate over metformin’s efficacy in kidney disease [19, 20]. Experimental data indicate that metformin modulates renal pathology through both adenosine monophosphate-activated protein kinase (AMPK)-dependent and AMPK-independent pathways, influencing inflammation, fibrosis, lipotoxicity, and autophagy [20].

Till now, this renoprotective action of metformin is scarcely proven in interventional clinical trials for CKD patients. Furthermore, no head-to-head randomized controlled trial was designed to compare this effect with SGLT2 inhibitors that might change the real-world practice for managing CKD [21]. According to the aforementioned knowledge, we conducted this study which aimed at evaluating the efficacy and safety of metformin versus empagliflozin, a SGLT2 inhibitor, on CKD progression with exploring proposed mechanistic intermediates in clinical settings.

## Patients and methods

### Study design and patients

This study was designed to be a 12-month multi-center, open-label, parallel-group, randomized controlled trial. Patients were recruited multi-centrally between January 2022 to April 2024 from the Urology and Nephrology Center, Mansoura University (two centers: Mansoura and Sammanoud cities) and Nephrology Outpatient Clinic, Internal Medicine Department, Tanta University Teaching Hospitals, Egypt.

**Inclusion criteria**: Patients with moderate CKD (stage 3: estimated glomerular filtration rate [eGFR] between 30 and 59 ml/min/1.73 m^2^) [11], aged ≥ 18 years, with and without type 2 diabetes (with no more than ten years of diabetes history for diabetics), with or without albuminuria, both genders are accepted. Patients on stable renin angiotensin system blockades dose, for at least a month before enrollment unless contraindicated, not tolerated, or not required based on clinical judgment, were also included. Renal functions should be stable in eligible patients for at least 3 months prior to enrollment.

#### Exclusion criteria:

Type 1 diabetics, patients with known hepatic cell failure, decompensated heart requiring acute management, planned surgical interventions, active malignancy, chronic inflammation, trauma, or infection on clinical bases. Pregnant or lactating women were excluded. Patients with known hypersensitivity to study medications, already on metformin or a SGLT2 inhibitor, or possessed any of study treatment labeled contraindications were also excluded.

Eligible patients were concealed randomized using restricted block randomization with reordering periodically change by third naïve independent coordinator with 1:1:1 ratio into three groups: *Group 1 (Control group)* who continued their standard therapy, *Group 2* who received metformin 500 mg/12hr orally after meals (Cidophage^®^ 500 mg Chemical Industries Development Company, Egypt) plus standard therapy, *Group 3* who received empagliflozin 10 mg once daily orally in the morning before meals (Mellitofix^®^ 10 mg, EVA Pharma Company, Egypt) plus standard therapy. Standard therapy referred to the evidence-based management of CKD, tailored according to relevant guideline recommendations, including blood pressure, glycemic, and lipid control with other supportive care as appropriate [22, 23]. Group 2 started with 500 mg metformin once daily for a week then titrated to the dose of 1000 mg for the next week till study end to minimize gastrointestinal side effects. At baseline, all participants were interviewed to a full medical and medication history with suitable clinical examination. Each patient was followed for 12 months, scheduled as monthly visits for the first six months then each trimester during the second six months to check drug adherence, safety, and other related therapeutic issues with clinical assessment and monitoring. Lifestyle, personal hygiene, adequate hydration and diet habits were educated and informed to all participants at baseline and their consistency were assessed and secured at each visit. Medications other than study treatments that could affect study outcomes were almost kept unchanged during follow-up period in all groups.

#### Ethical approval:

was taken prospectively from the Research Ethics Committee/Institutional Review Board of Tanta University (Code no.: 34976/10/21) and Mansoura University (Code no.: MDP.21.11.90). All study procedures obeyed the standards of the Declaration of Helsinki (1964) principles. Informed consent was taken from all eligible study patients before participation. The study was registered at clinicaltrial.gov (NCT05373680).

## Study outcomes

The primary outcome was the change in eGFR from baseline at study end with illustrating different effects regarding diabetes status. Secondary outcomes were assessed at baseline and after 12 months including the percent (%) changes in urinary albumin-to-creatinine ratio (uACR), TGF-β1, KIM-1, and beclin-1 levels. In addition to changes in glycated hemoglobin (HbA1c), body mass index (BMI), systolic and diastolic blood pressure (BP), uric acid level, lipid profile of total cholesterol (TC), triglycerides (TGs), high-density lipoprotein cholesterol (HDL-C), and low-density lipoprotein cholesterol (LDL-C). Drug tolerability and safety issues were monitored through study period.

## Assessment and biochemical measurements

Blood and urine samples were drawn from each participant while fasting in the morning of each visit to measure the laboratory parameters. All biological samples were coded and analyzed in a blinded manner to ensure unbiased assessment by the investigators. Serum creatinine was measured spectrophotometrically based on Jaffe reaction, to be used for calculating eGFR based on the 2021 Chronic Kidney Disease Epidemiology Collaboration (CKD-EPI) equation [11]. Urinary albumin and creatinine were assessed quantitively from fresh spot urine samples for calculating uACR. Whole blood samples were used to determine HbA1c levels. Spectrum kits (Egyptian Company for Biotechnology, Egypt) were used for the quantitative measuring of serum creatinine, HbA1c, urinary albumin and creatinine. Enzyme-Linked Immunosorbent Assay (ELISA) technique was used for assessing TGF-β1, KIM-1, and beclin-1 serologically using DLdevelop kits (Wuxi Donglin Sci & Tech Development Co., Ltd., China) (Catalog No.: DL-TGFb1-Hu, DL-KIM1-Hu, DL-BECN1-Hu, respectively). Serum uric acid and lipid parameters were assessed quantitively by enzymatic colorimetric assay using Spinreact kit (Spinreact, S.A/S.A.U. Company, Spain), while LDL-C was calculated using Friedewald’s Formula [24]. Weight and height were assessed for calculating BMI by dividing the weight (kg) by the squared height (m^2^). BP was measured in a seated five-minute resting position. Adverse events were recorded during the study follow-up period using the Medical Dictionary for Regulatory Activities (MedDRA) terminology for expressing the events. Close monitoring for symptoms of vitamin B12 deficiency and its serological levels were monitored particularly in the metformin group. Routine laboratory measurements of complete blood count (CBC), serum electrolytes, fasting blood glucose, liver enzymes, serum albumin and bilirubin were monitored each visit for safety assessment.

## Sample size calculation

Sample size was calculated based on the secondary renal outcomes of EMPA-REG trial that revealed an adjusted mean difference of eGFR 4.7 ml/minute/1.73 m^2^ from baseline to the study end of empagliflozin compared to placebo in diabetics [25]. Additionally, data from a retrospective large study conducted by Kwon S. et al. [26] revealed a comparative metformin effect on long term dichotomous hard renal outcomes in diabetic patients as shown in the EMPA-REG sub-analysis derived data. So, we hypothesized that metformin could exert an equivalent mean difference from control group for our primary outcome. By using G*Power 3.1.9.6 sample size calculator, and to achieve 80% power at two-sided significance level of 0.05, 18 patients were needed for each group. As we enrolled diabetic and non-diabetic patients, a duplicated sample was used to reach 36 patients per group. With an attrition rate of 10%, a total sample size of 120 patients was needed (40 patients per group).

### Statistical analysis

Statistical Package for the Social Sciences (SPSS) software program version 27.0.1 (IBM Corp., Armonk, NY, USA) was used for testing our data. Microsoft Office Excel 2019 (Microsoft Corp., USA) was used for data organization and graphical presentation. Continuous variables were first tested for normality using the Shapiro–Wilk and Kolmogorov–Smirnov tests. Normally distributed data were presented as mean ± standard deviation (SD) or standard error (SE) and analyzed using Paired-samples *t*-test or Analysis of Variance (ANOVA) followed by Tukey’s *post hoc* tests, as appropriate. Non parametric data were expressed as median (interquartile range [IQR]) and compared by Wilcoxon Signed-Rank and Kruskal–Wallis tests for paired and unpaired data, respectively. Categorical variables were summarized as frequencies and percentages and analyzed using the Chi-square test. Despite balanced baseline characteristics, residual effects of diabetes status, baseline eGFR and albuminuria could affect our primary outcome as stated in the *post hoc* EMPA-KIDNEY trial [27], so eGFR changes among groups were adjusted to baseline eGFR and uACR levels using univariate Analysis of Covariance (ANCOVA) with Bonferroni pairwise comparisons and then stratified based on the diabetes status. Skewed data of uACR, TGF-β1, KIM-1, and beclin-1 were log-transformed before analysis, then exponentiated to be expressed as geometric means to determine changes with baseline values adjustment in ANCOVA. Percent changes were calculated as (e^mean difference in log scale^−1)*100 and expressed as % change (95% confidence interval [CI]) [28, 29]. Spearman’s rank correlation was used for testing the correlation between biomarkers and renal parameters as pooled exploratory analyses. In all analyses, *p*˂0.05 inferred statistically significant.

## Results

**Fig. 1:**
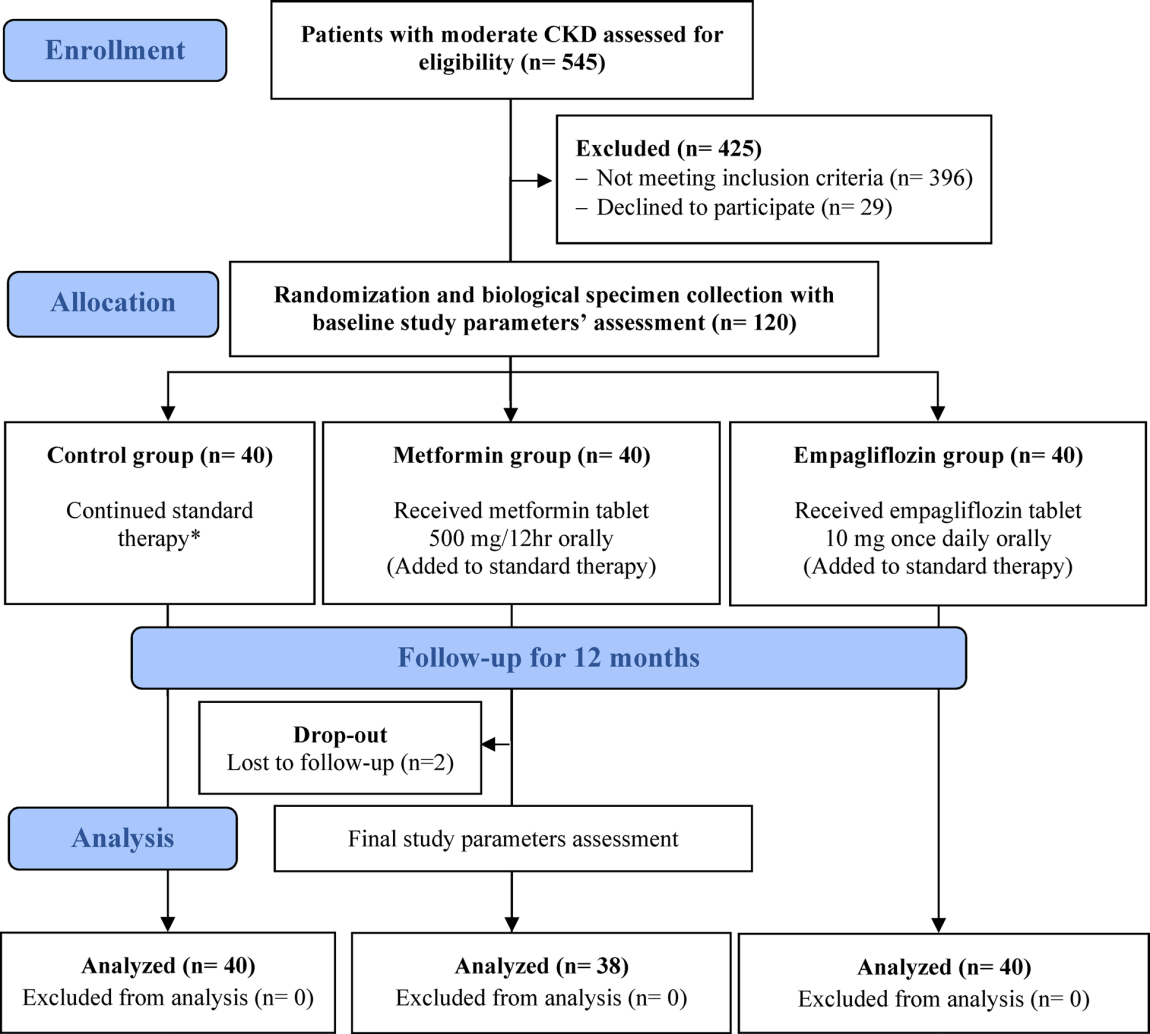
CONSORT flow diagram of the study participants. CKD: chronic kidney disease. *Referred to the evidence-based management of CKD, tailored according to relevant guideline recommendations, including blood pressure, glycemic, and lipid control with other supportive care as appropriate

A total of 120 eligible patients were randomly allocated, 118 patients out of them completed the full 12-month study period (Fig. 1). Baseline characteristics showed non-significant differences in all parameters as summarized in Table 1. Both empagliflozin and metformin were excellent adherent with the proportion of days covered (PDC) > 80% of required dose.

**Table 1:** Baseline characteristics of study patients

Parameter	Group 1 Control (*n* = 40)	Group 2 Metformin (*n* = 38)	Group 3 Empagliflozin (*n* = 40)	*p*-value
**Age (Years)**	53.5 (38.5–60)	59.5 (44.8–63.3)	53.5 (42.3–62)	0.23
**Sex: Female/Male (n)**	16/24	19/19	16/24	0.59
**Smoking (n/%)**	13 (32.5)	12 (31.6)	12 (30)	0.97
**History of diabetes (n/%)**	18 (45)	21 (55.3)	20 (50)	0.66
**History of hypertension (n/%)**	32 (80)	36 (94.7)	35 (87.5)	0.15
**SBP (mmHg)**	130 (130–140)	130 (120–140)	130 (130–130)	0.99
**DBP (mmHg)**	80 (80–80)	80 (80–90)	80 (70–90)	0.82
**Family history of CVD and/or CKD (n/%)**	17 (42.5)	11 (27.5)	14 (35)	0.46
**CKD disease duration (Years)**	1.75 (0.5–6.8)	2 (0.5–6.5)	1.75 (0.5–7.5)	0.99
**eGFR (ml/min/1.73 m** ^**2**^ **)**	42.3 ± 7.96	42.71 ± 8.67	42.75 ± 9.03	0.97
Non-diabetics Diabetics	43.64 ± 8.2 40.67 ± 7.55	46.29 ± 8.61 39.81 ± 7.74	43.4 ± 8.58 42.1 ± 9.63	0.52 0.68
**uACR (mg/g)**	220 (48.8–589)	222.5 (58.2–730.3.2.3)	306.05 (189–765.2.2)	0.27
**TGF-β1 (pg/ml)** **KIM-1 (pg/ml)** **Beclin-1 (ng/ml)**	2045.8 (1772.3–2880.6.3.6) 60.55 (54.6–90.1) 0.08 (0.01–0.21)	2426.5 (1992.8–2969.3.8.3) 107.15 (45.6–136.6.6.6) 0.09 (0.05–0.14)	2368.5 (2005–3189.5.5) 95.75 (68.3–124) 0.07 (0.02–0.15)	0.11 0.08 0.51
**HbA1c (%)** Non-diabetics Diabetics	5.33 ± 0.35 6.98 ± 2.09	5.62 ± 0.4 6.62 ± 1.07	5.51 ± 0.55 6.89 ± 1.91	0.12 0.79
**BMI (kg/m** ^**2**^ **)**	31.16 ± 6.62	33.86 ± 5.84	32.72 ± 6.25	0.16
**Uric acid (mg/dl)**	7.02 ± 1.86	7.08 ± 2.09	7.41 ± 1.78	0.62
**Lipids** TC (mg/dl) TGs (mg/dl) HDL-C (mg/dl) LDL-C (mg/dl)	183.13 ± 51.08 174 (148.3–210) 44.04 ± 9.45 103.71 ± 48.12	180.03 ± 41.68 170.5 (143.8–195.8.8.8) 42.8 ± 9.3 103.59 ± 38.55	171.44 ± 32.66 158.5 (130.5–188.8.5.8) 44.24 ± 11.2 92.39 ± 28.66	0.45 0.8 0.79 0.34
**Baseline comorbidities (n/%)** Cardiomyopathy/Heart failure Ischemic heart disease Arrhythmia	3 (7.5) 5 (12.5) 0	2 (5.3) 8 (21.1) 1 (2.6)	4 (10) 11 (27.5) 1 (2.5)	0.73 0.25 0.59
**Baseline medications (n/%)** Renin–angiotensin system inhibitor Beta-blocker Diuretic Calcium-channel blocker Any antihyperlipidemic Antiplatelet/anticoagulant Insulin DPP-4 inhibitors Sulfonylurea Any antihyperuricemics Antianginal	32 (80) 17 (42.5) 11 (27.5) 17 (42.5) 21 (52.5) 9 (22.5) 3 (7.5) 0 3 (7.5) 23 (57.5) 3 (7.5)	34 (89.5) 19 (50) 16 (42.1) 25 (65.8) 21 (55.3) 13 (34.2) 7 (18.4) 2 (5.3) 5 (13.2) 19 (50) 4 (10.5)	34 (85) 17 (42.5) 18 (45) 22 (55) 20 (50) 17 (42.5) 3 (7.5) 3 (7.5) 8 (20) 23 (57.5) 3 (7.5)	0.51 0.89 0.2 0.15 0.99 0.12 0.26 0.22 0.23 0.50 0.91

Data are expressed as mean ± SD or median (IQR) for continuous parametric or non-parametric data, respectively, and number (percent) for categorical data. SBP: systolic blood pressure; DBP: diastolic blood pressure; CVD: cardiovascular disease; CKD: chronic kidney disease; eGFR: estimated glomerular filtration rate; uACR: urinary albumin-to-creatinine ratio; TGF-β1: transforming growth factor-β1; KIM-1: kidney injury molecule-1; HbA1c: glycated hemoglobin; BMI: body mass index; TC: total cholesterol; TGs: triglycerides; HDL-C: high-density lipoprotein cholesterol; LDL-C: low-density lipoprotein cholesterol; DPP-4: dipeptidyl peptidase-4, *p<*0.05 inferred statistically significant

## Effects on eGFR changes

**Fig. 2:**
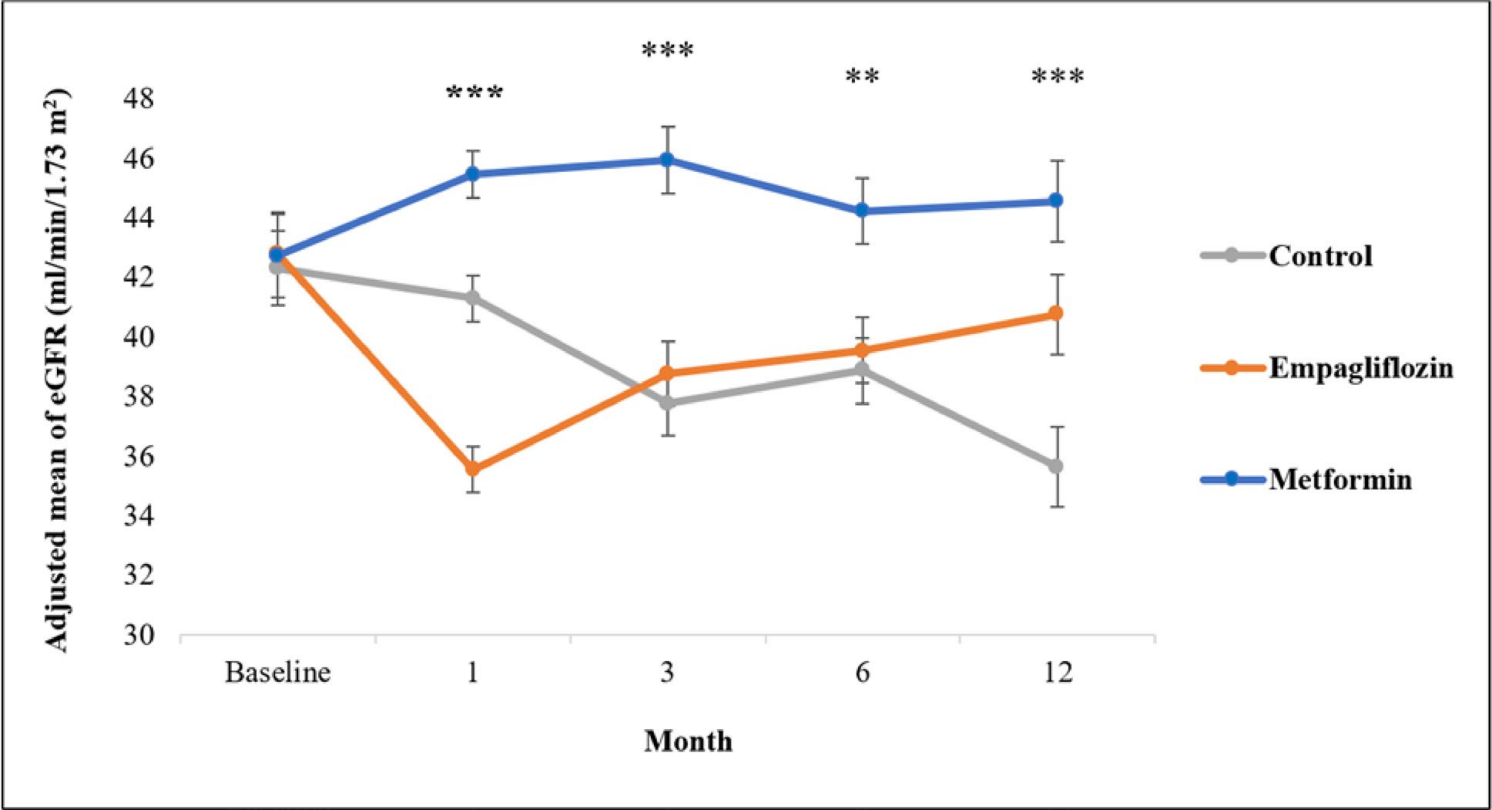
Trend changes in eGFR over 12-month follow-up period in pooled studied groups. Data are expressed as mean ± SE. eGFR: estimated glomerular filtration rate.** Independent *p*-value ˂ 0.01, ***Independent *p*-value ˂ 0.001, eGFR mean is adjusted to baseline eGFR and uACR levels

Figure 2 represent the trend of eGFR changes within the 12-month follow-up framework illustrating the advantageous changes in favor of metformin then empagliflozin treatment compared to control group. Empagliflozin therapy was initiated with eGFR decline at the first month that was resolved afterwhile. As regards to the primary outcome as summarized in Table 2, eGFR decreased significantly in the control group after 12 months without significant changes in other treatment groups. After stratification regarding diabetes, eGFR levels still decreased significantly in control group in both subgroups. The same was showed in non-diabetics in the empagliflozin group, without significant changes in empagliflozin treated diabetics or either subgroup after metformin therapy. By comparing among groups, data revealed significant improvement in eGFR in favor of 12-month metformin and empagliflozin therapy compared to the control group in all patients. After stratification, metformin’s effect was consistent in subgroups, while empagliflozin showed these favorable changes in diabetics only compared to the control group. Metformin therapy showed a greater improvement in eGFR than empagliflozin in non-diabetics, achieving borderline yet statistically significant difference (Table 2).

**Table 2:** Comparative effect of study groups on primary outcome, eGFR (ml/min/1.73 m^2^) changes, after 12 months with pairwise and subgroup analysis

Comparative groups/subgroups	Group 1 Control (*n* = 40)		Group 2 Metformin (*n* = 38)		Group 3 Empagliflozin (*n* = 40)	
	Baseline	After 12 months	Baseline	After 12 months	Baseline	After 12 months
***All patients***	42.3 ± 1.26	35.42 ± 1.73	42.71 ± 1.41	44.68 ± 1.73	42.75 ± 1.43	40.83 ± 1.92
P_1_-value	**˂ 0.001** ^*^		0.18		0.19	
***Diabetics***	40.67 ± 1.78	34.5 ± 2.51	39.81 ± 1.69	42.33 ± 1.59	42.1 ± 2.15	43.6 ± 2.94
P_1_-value	0.002*		0.22		0.53	
***Non-diabetics***	43.64 ± 1.75	36.18 ± 2.43	46.29 ± 2.09	47.59 ± 3.25	43.4 ± 1.92	38.05 ± 2.38
P_1_-value	**˂ 0.001** ^*^		0.55		**˂ 0.001** ^*^	

Post hoc analysis (adjusted mean difference)						*P*_2_-value
***All patients***						
Metformin vs. control	8.91 ± 1.92					**˂ 0.001** ^*^
Empagliflozin vs. control	5.1 ± 1.89					**0.03** ^*^
Metformin vs. empagliflozin	3.82 ± 1.92					0.15
***Diabetics***						
Metformin vs. control	9.11 ± 2.84					**0.007** ^*^
Empagliflozin vs. control	8.83 ± 2.89					0.01*
Metformin vs. empagliflozin	0.28 ± 2.74					**>** 0.99
***Non-diabetics***						
Metformin vs. control	8.59 ± 2.54					**0.004** ^*^
Empagliflozin vs. control	2.12 ± 2.41					**>** 0.99
Metformin vs. empagliflozin	6.47 ± 2.6					0.047*

Data were expressed as mean ± SE

eGFR: estimated glomerular filtration rate

P_1_**-**value: paired *p*-value (comparisons within group at baseline and after 12 months), P_2_-value: independent *p*-value (comparisons between groups), mean difference is adjusted to baseline eGFR and uACR using ANCOVA test with Bonferroni pairwise comparisons, *Significant difference (*p<*0.05)

## Effects on albuminuria, markers of fibrosis, kidney injury, and autophagy

**Fig. 3:**
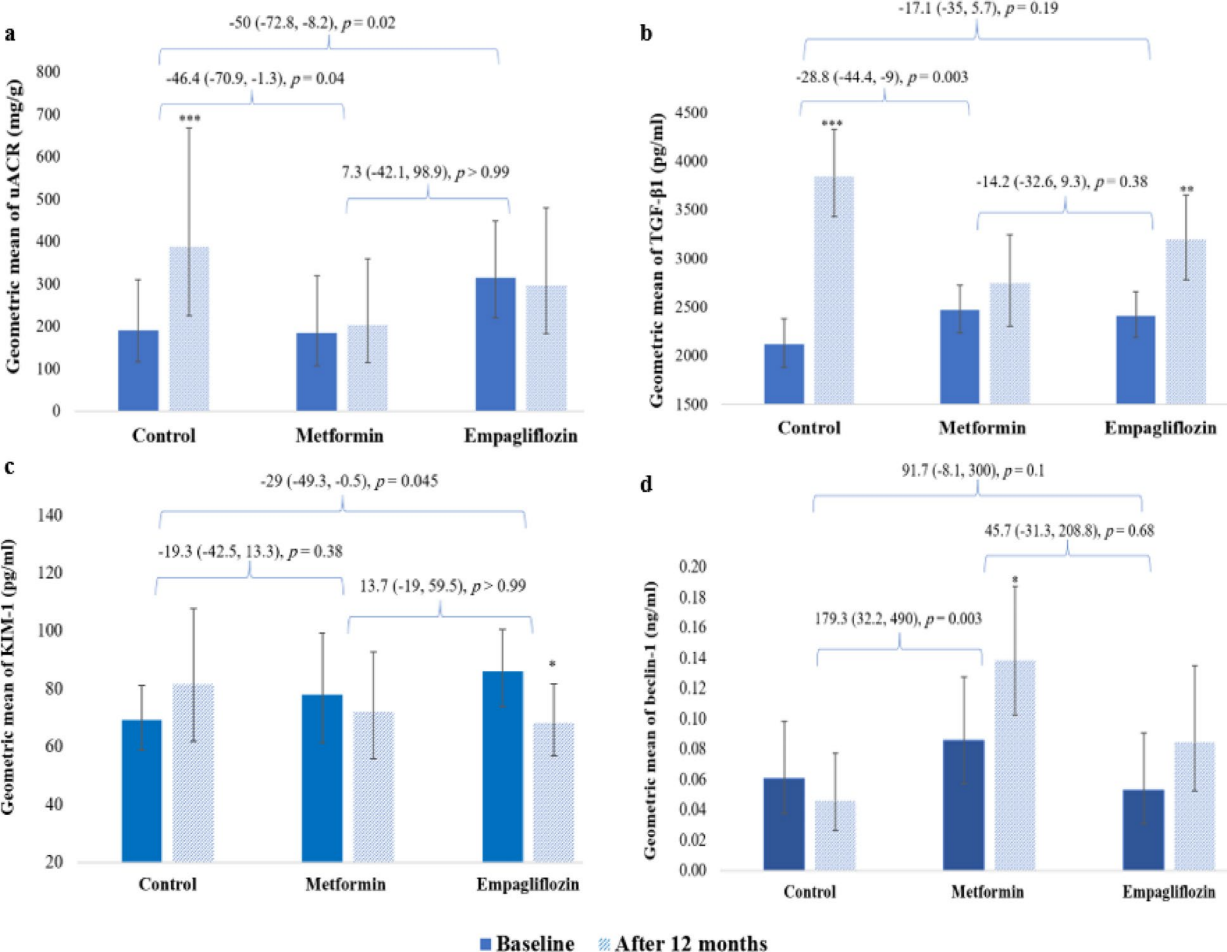
Effects of study groups on uACR, markers of fibrosis, kidney injury, and autophagy with their % relative changes. **a**: Geometric mean of uACR with % relative changes, **b**: Geometric mean of TGF-β1with % relative changes, **c**: Geometric mean of KIM-1with % relative changes, **d**: Geometric mean of beclin-1 with % relative changes. uACR: urinary albumin-to-creatinine ratio; TGF-β1: transforming growth factor-β1; KIM-1: kidney injury molecule-1. Bars represent geometric means and error bars represent their 95% confidence interval, *Significant difference within group (*p* ˂ 0.05), **Significant difference within group (*p* ˂ 0.01), ***Significant difference within group (*p* ˂ 0.001)

As illustrated in Fig. 3, uACR increased significantly in the control group (↑103.8% [95% CI: 41.1 to 194.4, *p* ˂ 0.001]) without being changed significantly after metformin and empagliflozin compared to their baseline values (↑9.7% [95% CI: −22.6 to 55.6, *p* = 0.59], ↓5.7% [95% CI: −35 to 36.7, *p* = 0.75], respectively). The comparison among groups revealed that both interventional treatments reduced uACR significantly compared to the control group without significant differences between them.

TGF-β1 levels increased significantly in the control and empagliflozin arms with highly pronounced increase shown in the control (↑81.3% [95% CI 56.2 to110.3, *p* ˂ 0.001] and ↑32.4% [95% CI 9 to 60.9, *p* = 0.006], respectively). Metformin didn’t induce significant change in the level of TGF-β1 (↑11% [95% CI −6.9 to 32.4, *p* = 0.24]). KIM-1 and beclin-1 levels were non-significantly deteriorated in the control group (↑18% [95% CI −5.9 to 48, *p* = 0.15], ↓25% [95% CI −55.8 to 27.1, *p* = 0.28], respectively). Treatment groups improved these levels with significant change in KIM-1 and beclin-1 from baseline in favor of empagliflozin and metformin (↓20.8% [95% CI −34.5 to −4.3, *p* = 0.02] and ↑62% [(95% CI 10.6 to 137.3, *p* = 0.02], respectively). When comparing among groups, data revealed non-significant differences expect for the protective effects exhibited by metformin on TGF-β1 and beclin-1 added to empagliflozin preserved effect on KIM-1 compared to the control group (Fig. 3).

### Effects on BMI, BP, and other metabolic parameters

As summarized in Table 3, HbA1c decreased significantly in both diabetics and nondiabetics by 12-month metformin therapy (mean difference ± SD: −0.4 ± 0.43%, *p* ˂ 0.001 and − 0.4 ± 0.3%, *p* ˂ 0.001, respectively). Empagliflozin decreased HbA1c significantly in diabetics without affecting it significantly in non-diabetics (mean difference ± SD: −0.89 ± 1.33%, *p* = 0.007 and − 0.22 ± 0.51%, *p* = 0.07, respectively). No significant differences were observed within control group and among groups. Similarly, BMI decreased significantly after 12-month metformin and empagliflozin therapy (mean difference ± SD: −0.72 ± 1.71 kg/m^2^, *p* = 0.01 and − 0.49 ± 0.99 kg/m^2^, *p* = 0.003, respectively), while it showed no significant changes within the control group and among groups. BP, either systolic or diastolic, showed non-significant variations within and among groups after 12-months follow-up period. Uric acid decreased significantly after 12-month empagliflozin therapy (mean difference ± SD: −1.09 ± 2.04 mg/dl, *p* = 0.002) without significant changes in either metformin or the control and even among groups. On the other hand, both 12-month metformin and empagliflozin decreased TC and LDL-C levels without affecting TGs or HDL-C significantly. All studied lipid parameters showed non-significant changes in the control group. The comparison among groups revealed significant differences in TC and LDL-C (*p* = 0.005 and 0.008, respectively) without significant differences regarding TGs and HDL-C. Post-hoc analysis illustrating the mean differences ± SE: −23.82 ± 8.76 mg/dl, *p* = 0.02 and − 19.33 ± 7.96 mg/dl, *p* = 0.04, for metformin versus control and − 25.78 ± 8.71 mg/dl, *p* = 0.01 and − 23.22 ± 7.86 mg/dl, *p* = 0.01, for empagliflozin versus control regarding TC and LDL-C, respectively. No significant differences were found between both interventional treatments.

**Table 3:** Changes in BMI, BP, and other biochemical parameters across study groups

Parameter	Group 1 Control (*n* = 40)		Group 2 Metformin (*n* = 38)		Group 3 Empagliflozin (*n* = 40)	
	Before	After 12 months	Before	After 12 months	Before	After 12 months
**HbA1c (%)** **Non-diabetics** **Diabetics**	5.33 ± 0.35 6.98 ± 2.09	5.34 ± 0.39 6.9 ± 1.79	5.62 ± 0.4 6.62 ± 1.07	5.25 ± 0.43^**a**^ 6.22 ± 1.01^**a**^	5.51 ± 0.55 6.89 ± 1.91	5.29 ± 0.53 6 ± 1.44^**a**^
**BMI (kg/m** ^**2**^ **)**	31.16 ± 6.62	31.33 ± 6.71	33.86 ± 5.84	33.14 ± 6.14^**a**^	32.72 ± 6.25	32.22 ± 6.16^**a**^
**SBP (mmHg)**	130 (130–140)	130 (120–140)	130 (120–140)	130 (130–130)	130 (130–130)	130 (122.5–140)
**DBP (mmHg)**	80 (80–80)	80 (80–90)	80 (80–90)	80 (80–80)	80 (70–90)	80 (80–80)
**Uric acid (mg/dl)**	7.02 ± 1.86	6.9 ± 1.72	7.08 ± 2.09	6.62 ± 2	7.41 ± 1.78	6.32 ± 1.85^**a**^
**TC (mg/dl)**	183.13 ± 51.08	183.45 ± 43.9	180.03 ± 41.68	159.63 ± 39.76^**a, b**^	171.44 ± 32.66	157.68 ± 31.29^**a, c**^
**TGs (mg/dl)**	174 (148.3–210)	187 (149.3–233.8.3.8)	170.5 (143.8–195.8.8.8)	162 (120–188)	158.5 (130.5–188.8.5.8)	156.5 (130–183.5.5)
**HDL-C (mg/dl)**	44.04 ± 9.45	44.25 ± 9.13	42.8 ± 9.3	43.34 ± 11.83	44.24 ± 11.2	45.26 ± 13.2
**LDL -C (mg/dl)**	103.71 ± 48.12	102.17 ± 41.24	103.59 ± 38.55	82.84 ± 33.67^**a, b**^	92.39 ± 28.66	78.96 ± 29.42^**a, c**^

Data were expressed as mean ± SD and median (IQR) for parametric and non-parametric continuous variables, respectively, HbA1c: glycated hemoglobin; BMI: body mass index; SBP: systolic blood pressure; DBP: diastolic blood pressure; TC: total cholesterol; TGs: triglycerides; HDL-C: high-density lipoprotein cholesterol; LDL-C: low-density lipoprotein cholesterol, ^a^ Significant difference within group, ^b^ significant difference between groups (metformin versus control), ^c^ significant difference between groups (empagliflozin versus control)

### Correlation analysis

**Fig. 4:**
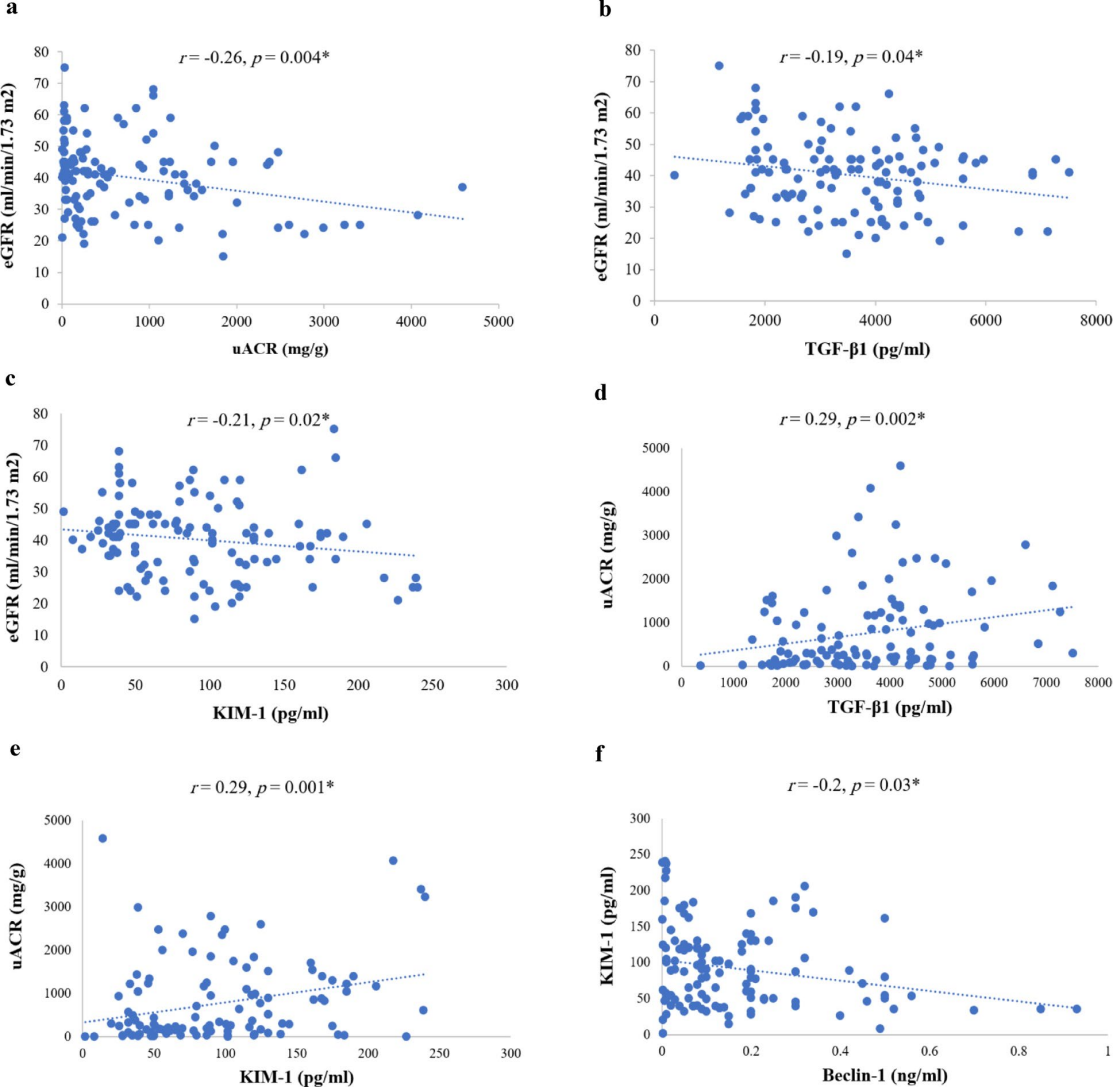
Significant pooled correlation for studied biomarkers after 12-month study period (*n* = 118). **a**: Correlation between eGFR and uACR; **b**: Correlation between eGFR and TGF-β1; **c**: Correlation between eGFR and KIM-1; **d**: Correlation between uACR and TGF-β1; **e**: Correlation between uACR and KIM-1; **f**: Correlation between KIM-1 and beclin-1; *r*: spearman’s correlation coefficient; eGFR: estimated glomerular filtration rate; uACR: urinary albumin-to-creatinine ratio; TGF-β1: transforming growth factor-β1; KIM-1: kidney injury molecule-1, *Statistically significant (*p<*0.05)

Pooled data after 12-month study period revealed significant inverted correlations between eGFR and (uACR, TGF-β1, and KIM-1), added to KIM-1 and beclin-1. Significant positive correlations were also demonstrated between uACR and (KIM-1 and TGF-β1) (Fig. 4).

### Safety and adverse events

**Fig. 5:**
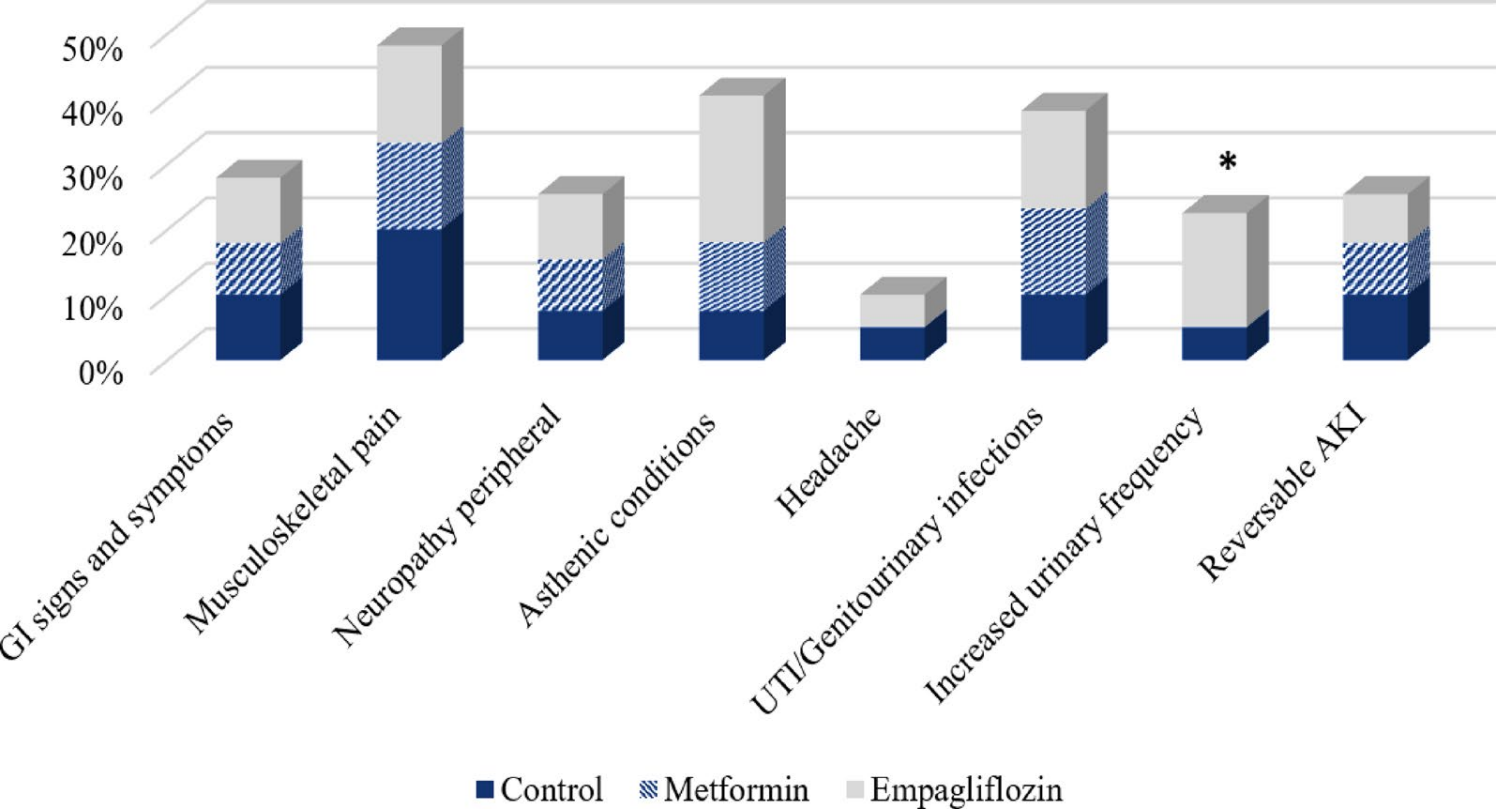
Recorded adverse events in studied groups. GI: gastrointestinal; UTI: urinary tract infection; AKI: acute kidney injury, *Significant difference among groups (*p<*0.05)

Safety issues were comparable among the three study groups without observed significant differences except for a tolerable increased urinary frequency in favor of empagliflozin group without documented volume depletion (Fig. 5). Changes in the levels of electrolytes, hemoglobin, hematocrit, albumin, bilirubin, liver enzymes revealed non-significant differences among the study groups (Supplementary Table). Neuropathy incidents were equivalent among groups without proven reported cases of metformin induced vitamin B12 deficiency. No cases of hypoglycemia, lactic acidosis, or ketoacidosis were recorded during the follow-up period in all study groups.

## Discussion

Preventing the progression of CKD is challenging with limited renoprotective treatment options. In this interventional clinical study, the renoprotective effect of metformin was evaluated in moderately CKD patients and compared with peers across empagliflozin and control arms with other metabolic and supposed mechanistic outcomes. During the current study, metformin dosed as 1000 mg daily based on the recommended dose for eGFR 30–44 ml/min/1.73 m^2^ [23]. Empagliflozin 10 mg daily dose was used as it is the acceptable and sufficient dose for renoprotection regardless diabetes [16, 27]. The study duration was 12 months which seems acceptable to allow the detection of early renal functional changes in moderately CKD patients with preserving patient’s adherence and retention, consistence with published specified analyses in similar CKD patients [27, 30].

Our primary outcomes demonstrated that 12-month metformin therapy increased eGFR significantly compared to control group with consistent beneficial effects between diabetics and non-diabetics. Empagliflozin treatment showed transient decline in eGFR in the first month then it lowered the decline in eGFR on long term therapy compared to control group with significant apparent effects in diabetics. Metformin was superior than empagliflozin in terms of non-diabetics, without being significantly different from each other in pooled or diabetic subgroups. Given the relatively small subgroup sizes with borderline significant difference, these findings are considered hypothesis-generating warranting confirmation in larger studies. Indeed, our results are agreed with Corremans R, et al., who demonstrated the equally renoprotective effects of metformin and canagliflozin, a SGLT-2 inhibitor, in diabetic CKD rat models [31]. In another short period preclinical study on non-diabetic rats, metformin showed significant improving in creatinine clearance with less sounded effects exhibited by empagliflozin [32]. In the context of clinical trials, the renoprotective effects of SGLT2 inhibitors were proven in large scaled clinical trials as DAPA-CKD [15], EMPA-KIDNEY [16], CREDENCE [33], EMPA-REG [25], while these trials focused on hard renal clinical outcomes with eGFR monitoring being tertiary or explanatory outcomes. Our empagliflozin results are consistence with post hoc of DAPA-CKD [30], EMPA-KIDNEY [27], and recent meta-analysis evaluated the cardiorenal protective effects of SGLT2 inhibitors including their large trials [14]. SGLT2 inhibitors from these data demonstrated an eGFR decline for the first few weeks followed by sustained improvement on long terms with more pronounced effects in diabetics. In contrary, clinical research intended to measure the favorable effect of metformin on kidney and its ability to improve eGFR in CKD patients is rare. Our former findings come in alignment with the recent large retrospective clinical trials demonstrated the proposed favorable effects of metformin on clinical renal outcomes [26, 34–36]. From those, study by Agur et al. demonstrated the addition metformin to a SGLT inhibitor possessed favorable actions than a SGLT alone [35]. Notably, these trials focused only on diabetics, forced the need for further large trials prove our results in non-diabetics too.

In our control group, albuminuria, markers of fibrosis, autophagy, and tubular injury were deteriorated by the study end which revealed disease progression particularly in levels of uACR and TGF-β1. Metformin therapy halted this deterioration with significant improvements in favor of TGF-β1 and beclin-1 levels compared to the control arm. These findings suggest that metformin exhibits renoprotective benefits through molecular mechanistic pathways beyond its glycemic control properties which comes in accordance with previously reported work. They summarized the presence of metformin in renal cell is associated with AMPK activation leading to subsequent cascaded pathway of induced autophagy with other anti-fibrotic and anti-inflammatory properties for kidney protection [19, 20]. The effect of metformin on TGF-β1 is also consistent with other previous preclinical studies [37, 38]. Additionally, metformin’s renoprotection via the antifibrotic properties can be proven by the significant correlation between eGFR and TGF-β1 levels observed during the current study. Regarding KIM-1, our findings are consistence with the results reported by Wang et al. who demonstrated the non-significant effect of metformin on urinary KIM-1 levels after 12 months in autosomal dominant polycystic kidney disease patients [39]. Conversely, Zhang et al. reported a significant reduction of serum KIM-1 in AKI mice models [40]. These controversial data sparked the need for clinical illustration of such effects in different disease states.

Indeed, the renoprotective effect of SGLT2 inhibitors was linked previously to their action on the tubule-glomerular feedback pathway [17, 18]. It may be returned to the pronounced reduction of eGFR decline in diabetics relative to non-diabetics even that beneficial clinical outcomes are preserved for both subgroups. The exact effect is still inconclusive with proposed other intermediate mechanistic cascades [41]. Our findings revealed favorable effects of empagliflozin on TGF-β1, KIM-1, and beclin-1 levels without significant difference relative to the control group except for KIM-1. It exhibited approximately 29% relative reduction in KIM-1 levels. This effect is consistence with results obtained by CANVAS trial demonstrated that canagliflozin produced a 26.7% relative reduction in KIM-1 as compared to placebo in diabetics with cardiovascular risk [29]. Additionally, a sub-analysis of the CardioLink-6 trial showed a 34% reduction in KIM-1 after a 6-month treatment with empagliflozin compared with placebo group in diabetic patients [42]. While, the effect of SGLT2 inhibitors on fibrosis and autophagy in kidney diseases was mainly reported by in-vitro and animal studies with controversial results [43–47]. This variability may be attributed to the difference in disease nature or research methodology.

Albuminuria in CKD patients originates from glomerular and/or tubular dysfunctions which is usually linked to cardiorenal progressive warnings [12, 48]. SGLT2 inhibitors reported favorable effects on reducing albuminuria from their large trials or further *post hoc* analysis [49–51]. They demonstrated significant reductions in uACR by 18%, 35.1%, and 31% for empagliflozin, dapagliflozin, and canagliflozin, respectively. More recently, the *post hoc* analysis of EMPA-KIDNEY revealed a range of 5–26% reductions by the implication of empagliflozin compared to placebo group based on the grade of baseline albuminuria [27]. These results are consistence with our findings as empagliflozin achieved a 50% reduction in uACR more than the control group. Similarly, metformin reduced uACR by 46% relative to our control group. Pan et al. previously demonstrated the significant comparative uACR reduction by 48-week metformin therapy in patients with low-grade albuminuria to non-albuminuric peers [52]. In contrary, metformin did not affect the incidence of albuminuria in diabetic population without renal disease according to recent large retrospective study [36]. This debate sparks the proposed variability of metformin’s effect regarding the baseline albuminuria that warrants further investigation. Preclinical studies revealed that metformin could reduce albuminuria via anti-fibrotic, anti-oxidant, anti-inflammatory pathways in the presence or absence of diabetes [53]. This aligns with our findings demonstrated favorable effects of metformin on markers of fibrosis and autophagy. On the other hand, empagliflozin could exhibit its effect through the tubulo-glomerular feedback and its favorable effect on tubular injury achieved by decreasing KIM-1 levels.

Previous observational studies on diabetics demonstrated controversial effects of metformin on uric acid levels [54, 55]. Our findings revealed a non-significant effect of metformin on serum urate. This result comes into alignment with Hsu et al. work, who assessed the renal effect of metformin retrospectively in moderately CKD diabetic patients and could not confirm its lowering effect on uric acid levels [56]. EMPA-KIDNEY *post hoc* analysis showed a modest decrease in uric acid after 18-month empagliflozin therapy with pronounced effects in higher eGFR and non-diabetics irrespective to its renoprotective action [57]. In our study, a 12 months treatment with empagliflozin reduced uric acid levels compared to baseline data but without significant difference compared to control group. This variation may be attributed to the difference in patients’ characteristics or the insufficient sample size.

Regarding lipid effects, both interventional drugs reduced TC and LDL-C levels relative to control which highlight their possible atheroprotective effects particularly in high-risk CKD patients. These effects could support treatments’ renoprotective action rather than being directly incorporated to their renal effects. The favorable effect of metformin on lipid profile was demonstrated before, particularly in diabetics, elucidating the AMPK-mediated pathways [58–60]. SGLT2 inhibitors showed contrary results with lack of extensive data regarding their effect on lipid parameters in CKD patients [61–64]. Importantly, these metabolic and cellular effects are closely associated with improved endothelial function, a key mechanism contributing to cardiovascular and renal complications in CKD, thereby potentially reducing disease-related morbidity and mortality [4, 65]. These results are consistent with recent evidence demonstrating the multi-faceted protective mechanisms of metformin and empagliflozin, including metabolic regulation, anti-inflammatory activity, and endothelial function modulation [59]. Further randomized controlled trials are needed to confirm these data with ensuring the exact mediated mechanistic pathways.

As they are antidiabetics, both empagliflozin and metformin significantly reduced HbA1c by the end of the study. Notably, metformin lowered also the HbA1c in nondiabetic subgroup without triggering hypoglycemia. This antidiabetic effect may be related to its beneficial effect on preserving prediabetes from diabetes [66–68]. However, the overall effect of both treatments on glycemic issues was not differed significantly when compared to the control group. This can be explained as our study diabetic groups were already on antidiabetic therapy and showed generally a good glycemic control at baseline. The same was noticed for the effects of both medications on body weight, as they resulted in BMI reductions without being significantly differed from the control group. Moreover, blood pressure did not change significantly in all study groups. Empagliflozin was previously reported to possess mild effects on body weight and blood pressure [12, 69]. Supposed mechanism is shifted towards its glycosuria-induced low caloric and natriuresis which was not necessarily being statistically significant. Similarly, metformin was reported to produce about 1–5 kg weight reduction in diabetics with limited data regarding CKD patients [70, 71]. Its effect on BP is controversial, tending towards being modest and non-significant particularly for diastolic [72–74]. Concerning safety issues, the implication of empagliflozin and metformin for 12 months for patients with moderately CKD was safe, tolerable, and didn’t provoke serious adverse effects. Just tolerable increased urination frequency was reported more in empagliflozin therapy, linked to its mechanism of action, while not exacerbating hypovolemia or drug noncompliance. This finding comes in accordance with many previous studies indicated safety and tolerability of such drug [16, 25, 26].

The points of strength of the current study include its design as a randomized controlled parallel study, being multicenter study, the good study duration relative to its main objectives, and its priority as a first clinical study aimed at assessing the renoprotective effect of metformin, added to be compared with a SGLT2 inhibitor, according to the best of authors’ knowledge. Nevertheless, the current study has some limitations including a relatively small sample size and being open-label study. With the reliance mainly on objective assessments, concealed randomization, blinded laboratory analyses, and standardized follow-up procedures, the internal validity of the study results could be supported with bias minimization. Additionally, the primary outcome was priory powered for the overall CKD population, whereas subgroup analyses and secondary outcomes could be underpowered, thus they provide hypothesis-generating insights. This study also evaluated the renoprotective effects of the investigated agents in Egyptian patients with moderate CKD over a 12-month period, with particular focus on mechanistic pathways that remain not fully elucidated. In this context, future large-scale clinical trials involving more diverse populations are warranted to validate these findings, clarify the underlying mechanisms, and to guide evidence-based approaches addressing the global CKD burden.

## Conclusion

A 12-month treatment with metformin therapy produced a renoprotective effect in patients with moderate CKD with a head-to-head effect in comparison with empagliflozin therapy. It showed beneficial effects for halting CKD progression in both diabetics and nondiabetics with a greater protection than empagliflozin in non-diabetics. Metformin’s renal effects were linked to its antifibrotic and favorable autophagy effects. Empagliflozin proposed renoprotective effect is related to the tubulo-glomerular feedback with tubular injury protection. Both drugs reduced albuminuria and improved other metabolic parameters. Safety issues were generally comparable across study groups.

### Recommendations

This study opens the avenue for new era of managing CKD by repurposing metformin. Large scaled and double blinded randomized controlled trials with longer follow-up periods which could explore further hard cardio-renal clinical outcomes of metformin in patients with CKD are necessary. Future mechanistic studies, including experimental and molecular analyses, are also warranted to provide deeper insights into these renoprotective effects. Such effects warrant further investigation across different subgroups, diabetic statuses, broader CKD stages, and more diverse populations to strengthen the external validity of our findings. In addition, future studies with adequate sample sizes are needed to better delineate treatment-specific mechanistic correlation patterns. If all these considerations be confirmed, metformin could potentially challenge CKD treatment guidelines, as it would spark renoprotection with relatively lower cost and trivial adverse effects.

## Supplementary Information

Supplementary Material 1

## Data Availability

The data may be acquired from the corresponding author on reasonable request.

## Abbreviations

AMPK: Adenosine monophosphate-activated protein kinase
ANCOVA: Analysis of Covariance
ANOVA: Analysis of Variance
BMI: Body mass index
BP: Blood pressure
CBC: Complete blood count
CI: Confidence interval
CKD: Chronic kidney disease
CKD-EPI: Chronic Kidney Disease Epidemiology Collaboration
CVD: Cardiovascular disease
DBP: Diastolic blood pressure
DPP-4: Dipeptidyl peptidase-4
eGFR: Estimated glomerular filtration rate
ELISA: Enzyme-Linked Immunosorbent Assay
HDL-C: High-density lipoprotein cholesterol
HgA1c: Glycated hemoglobin
IQR: Interquartile range
KIM: Kidney injury molecule
LA: Lactic acidosis
LDL-C: Low-density lipoprotein cholesterol
MedDRA: Medical Dictionary for Regulatory Activities
SBP: Systolic blood pressure
SD: Standard deviation
SE: Standard error
SGLT2: Sodium-glucose cotransporter-2
SPSS: Statistical Package for the Social Sciences
TC: Total cholesterol
TGF-β1: Transforming growth factor-β1 (TGF-β1)
TGs: Triglycerides
uACR: Urinary albumin-to-creatinine ratio
