# Supplementary material for: Efficacy and safety of metformin versus empagliflozin on chronic kidney disease progression (MET-EMPA-CKD): a randomized controlled trial
Source: Diabetol Metab Syndr. 2025 Dec 10;18:17. doi: 10.1186/s13098-025-02040-9 (PMC12801430; doi:10.1186/s13098-025-02040-9)
Supplement: Supplementary file 1 — Supplementary Material 1 [file 13098_2025_2040_MOESM1_ESM.docx]

- **Article Title:** Efficacy and Safety of Metformin Versus Empagliflozin on Chronic Kidney Disease Progression (MET-EMPA-CKD): A Randomized Controlled Trial
- **Journal:** Diabetology & Metabolic Syndrome
- **Authors:** Bassant M. Mahboub, Ayman F. Refaie, Sahar M. El‑Haggar, Yasser M. Hafez, Tarek M. Mostafa
- **Corresponding author:**

Bassant Maher Mahboub

Drug and Poison Information Center Director, Faculty of Pharmacy, Tanta University, Tanta 31527, Egypt

Clinical Pharmacy and Pharmacy Practice Department, Faculty of Pharmacy, Mansoura National University, Gamasa 7731168, Egypt

E-mail: [bassantm.mahboub@pharm.tanta.edu.eg](mailto:bassantm.mahboub@pharm.tanta.edu.eg%20) , Mob.: +201205448976

**Supplementary Table** Changes in the levels of electrolytes, hemoglobin, hematocrit, albumin, bilirubin, and liver enzymes across study groups

| **Parameter** | **Group 1**  **Control (n= 40)** | | **Group 2**  **Metformin (n= 38)** | | **Group 3**  **Empagliflozin (n= 40)** | |
| --- | --- | --- | --- | --- | --- | --- |
|  | **Before** | **After  12 months** | **Before** | **After  12 months** | **Before** | **After  12 months** |
| **Potassium (mmol/L)** | 4.52 ± 0.39 | 4.5 ± 0.58 | 4.37 ± 0.45 | 4.39 ± 0.41 | 4.34 ± 0.42 | 4.3 ± 0.56 |
| **Sodium (mmol/L)** | 138 (3) | 138 (3) | 137 (2) | 137.5 (2) | 137 (3) | 137 (2)^a^ |
| **Calcium (mg/dL)** | 9.85 (0.9) | 9.55 (0.85)^a^ | 9.95 (0.7) | 9.69 (0.37)^a^ | 9.8 (0.78) | 9.69 (0.88) |
| **Phosphorus (mmol/L)** | 3.53 ± 0.66 | 3.75 ± 0.6 | 3.58 ± 0.6 | 3.79 ± 0.74 | 3.57 ± 0.52 | 3.59 ± 0.56 |
| **Albumin (g/dL)** | 4.1 (0.4) | 4.1 (0.5) | 4.1 (0.6) | 4.2 (0.6) | 4 (0.5) | 4 (0.4) |
| **Total Bilirubin (mg/dL)** | 0.48 (0.1) | 0.47 (0.08) | 0.48 (0.17) | 0.5 (0.18) | 0.47 (0.09) | 0.44 (0.06) |
| **ALT (U/L)** | 19.55 ± 9.45 | 20.3 ± 10.31 | 17.79 ± 6.62 | 18.71 ± 5.64 | 19.95 ± 6.94 | 20.33 ± 7.57 |
| **AST (U/L)** | 21.68 ± 6.8 | 22.7 ± 6.71 | 21.68 ± 8.14 | 22.71 ± 6 | 22.83 ± 6.09 | 22.13 ± 6.86 |
| **Hemoglobin (g/dL)** | 13.28 ± 1.67 | 13.06 ± 2.21 | 12.5 ± 1.47 | 12.59 ± 1.72 | 12.9 ± 1.79 | 13.12 ± 1.99 |
| **Hematocrit (%)** | 42.31 ± 5.2 | 41.45 ± 6.52 | 40.22 ± 4.21 | 39.22 ± 5.31 | 40.3 ± 5.05 | 41.08 ± 5.62 |

Data were expressed as mean ± SD and median (IQR) for parametric and non-parametric continuous variables, respectively

ALT: alanine aminotransferase; AST: aspartate aminotransferase

^a^ Significant difference within group (*p*˂0.05)
